# Relationship Between Online Health Information Acquisition and Shared Decision-Making Among Patients With Diabetes: Cross-Sectional Survey Study

**DOI:** 10.2196/86137

**Published:** 2026-04-30

**Authors:** Yue Ming, Miriam L Matteson, Yao Zhang

**Affiliations:** 1 Rudolph Matas Library of the Health Sciences, Tulane University, New Orleans, LA, United States; 2 College of Communication and Information, Kent State University, Kent, OH, United States; 3 Department of Information Resource Management, School of Information and Communication, Nankai University, 38 Tongyan Road, Jinnan District, Tianjin, 300350, China, 86 22-23506326

**Keywords:** online health information acquisition, active online health information seeking, incidental online health information acquisition, eHealth literacy, shared decision-making, health communication, diabetes mellitus

## Abstract

**Background:**

Shared decision-making allows patients and clinicians to make decisions together to help determine the most appropriate option. Patients need comprehensive health information to participate and evaluate different options during the shared decision-making process. Patients with diabetes need to constantly monitor their health status. They experience an array of health information needs during their ongoing health management. Online health information acquisition is a common behavior among patients with diabetes, and online information can impact the interaction between patients with diabetes and health care providers.

**Objective:**

This study explored the relationship between 2 types of online health information acquisition behavior (active online health information seeking and incidental online health information acquisition) and shared decision-making. It also investigated the mediating role of eHealth literacy during the information acquisition process among US patients with diabetes aged 18 to 44 years.

**Methods:**

Participants were patients with diabetes aged 18 to 44 years in the United States and were recruited by a survey company, Centiment. The sampling process matched the national distribution of gender and age in the United States. An online survey questionnaire was distributed through Qualtrics. A total of 558 valid responses were collected. The average age of the sample was 35.91 (SD 6.04) years. Among the sample, 260 participants were men, 291 participants were women, and 7 participants identified their gender as other. Bivariate analyses and partial least squares structural equation modeling were used for data analysis. All data analyses were performed in R.

**Results:**

The prevalences of active online health information seeking (mean 3.97, SD 0.78) and incidental online health information acquisition (mean 4.27, SD 0.78) were high among participants. Education was a key factor related to eHealth literacy (*P*<.001) and shared decision-making (*P*<.001). Model testing indicated that active online health information seeking was related to eHealth literacy (*β*=.192, 95% CI .067-.320) and shared decision-making (*β*=.234, 95% CI .123-.346). Incidental online health information acquisition was related to eHealth literacy (*β*=.335, 95% CI .205-.461). eHealth literacy was related to shared decision-making (*β*=.441, 95% CI .334-.536). Therefore, eHealth literacy partially mediated the relationship between active online health information seeking and shared decision-making, while it fully mediated the relationship between incidental online health information acquisition and shared decision-making.

**Conclusions:**

This study contributes to the ongoing development of health communication strategies and the modification of health information training programs for patients with diabetes. The need for the information industry to deliver accurate and easy-to-understand health information to the public to support their decision-making process and encourage positive health behaviors is urgent.

## Introduction

### Background

Engaging patients in the medical decision-making process has become a common practice to increase patients’ self-care adherence and improve their wellness [1,2]. Shared decision-making is a decision-making process involving both physicians and patients [3]. The process allows patients and clinicians to make decisions together to help determine what is the most appropriate option for each patient. The successful implementation of such a process requires health practitioners to share knowledge and patients to consent to the process. More importantly, shared decision-making encourages patients to feel confident in making decisions based on their own circumstances [4]. To take full advantage of the shared decision-making process, patients need comprehensive information to participate in the process and to evaluate different options [5,6]. For example, depending on different health conditions, patients may need time to process new information (eg, new treatments, drug effects) and then take into consideration their personal preferences (eg, affordability, additional underlying health conditions) [7,8]. A few studies have investigated the relationship between information behavior factors and shared decision-making from various perspectives. In a meta review aimed at identifying the influences on information exchange in shared decision-making, researchers argued that health literacy mediates the relationship between information exchange and the shared decision-making process [9]. Other scholars have suggested that during the shared decision-making process, patients may access health information through both health providers’ digital channels and external sources [10,11].

With the rapid development of information technology, the internet serves as an important source of health information [12,13]. Researchers have illustrated that young adults tend to use online technologies more actively than the older generation [14]. However, the availability of online health information might not necessarily contribute to better understanding and use of health information [15,16]. Key information practices such as accumulating knowledge, staying well informed, and using information to make decisions are the foundations of eHealth literacy and are critical for effective information seeking and use. Based on the unique characteristics of the online environment, eHealth literacy has become a focal point for researchers studying online information-seeking behavior. eHealth literacy extends various aspects of traditional information literacy to focus on an individual’s ability to seek, process, and interpret health information in the online environment [17]. Researchers have suggested that eHealth literacy is associated with online health information-seeking behaviors within different health contexts [18,19]. Moreover, eHealth literacy has also been identified as a key factor related to various health behaviors and health outcomes [20-22].

Information seeking refers to the process of people looking for information to fulfill a purpose or solve a problem [23]. The phrase “information acquisition” provides a broader umbrella to include purposeful information seeking and passive information encountering [24,25]. The ecological model of information use suggests that both active information seeking and incidental information acquisition can occur during our everyday lives [26]. More specifically, active information seeking refers to people purposefully seeking and obtaining information to address problems, while incidental information acquisition refers to people’s passive consumption of information from exposure to information sources. Furthermore, when users intentionally seek information that they know they need, they may also unintentionally collect other information they encounter during the information-seeking process, though they do not realize that they may need it. Thus, both types of information acquisition behavior can occur simultaneously, especially within the current digital age, due to the ease of obtaining information online.

The framework of “searching as a learning process” demonstrates the intertwined nature of searching and learning [27]. It suggests that people acquire knowledge critically and receptively through iterative and reflective search behaviors. During this process, comprehensive information skills, such as analyzing, evaluating, and appraising information, can also be learned and improved [28]. Since the model of the information search process [29] demonstrated that information seeking commonly involves knowledge construction and learning, it is important to note that similarly, people can also learn and improve their comprehensive information skills during the process of online health information seeking. Furthermore, online health information acquisition can not only contribute to the knowledge acquisition process but also increase the level of exposure to technology and the internet for health information. Previous studies have demonstrated that exposure, such as the frequency of use of internet and web searches regarding health information, is positively related to eHealth literacy [30,31]. Researchers have also identified the mediating role of eHealth literacy during the process of online information seeking and doctor-patient interaction in China [32]. Specifically, eHealth literacy was one of the mediators that fully and positively mediated the association between online health information-seeking behavior and doctor-patient interactions.

### Objectives

Chronic diseases require long-term self-care in both medical and everyday life. People living with chronic diseases need to constantly collect useful information to adjust their behaviors. Diabetes is one of the most common chronic diseases and is a serious public health problem around the world. Data suggest that about 589 million adults worldwide had diabetes in 2024, with a projection that the number would rise to about 853 million by 2050 [33]. Scholars have also illustrated that the rate of diabetes is increasing significantly among young adults [34]. Diabetes progresses through various stages that involve various types of treatments. In addition to medical treatment, patients with diabetes must follow recommended lifestyle practices to slow the decline in their health [35]. Patients with diabetes can decide which treatments or approaches are the best for them. The process of making such decisions may not relate to medical conditions or clinical recommendations but may also involve other factors, such as costs, side effects, and other specific individual contexts [36-38]. Patients with diabetes need to constantly monitor their health status to keep the disease under control and prevent it from progressing to more complicated stages. This constant monitoring suggests that patients with diabetes experience an array of health information needs during their ongoing health management. By obtaining such information, people living with diabetes can exchange opinions with health care providers and adjust their behaviors accordingly. However, when further examining the impacts of health information acquisition on users’ health behaviors in the context of diabetes, researchers [39-42] tend to focus on either active information seeking or incidental information acquisition rather than investigating both behaviors at the same time. It is also important to note that the information sources for both health information acquisition behaviors can vary significantly. Therefore, the cognitive process following active information seeking or incidental information acquisition might differ due to the distinct levels of individuals’ perception of various information sources. As a result, effects from both online health information acquisition behaviors might be reflected distinctively in health behaviors through eHealth literacy. Moreover, there is a lack of research that examines shared decision-making through the perspective of information acquisition or observation of patients’ information acquisition behaviors. It is necessary to extend the understanding of the links between different patterns of health information acquisition behaviors and shared decision-making to promote effective health communication strategies and online health information consumption skills. Additionally, since only a few studies have investigated the role of eHealth literacy during the shared decision-making process, little is known about the possible roles of eHealth literacy in the link between information acquisition and shared decision-making. Data from the Pew Research Center suggest that young adults tend to use online technologies more actively than older generations [14]. Furthermore, the prevalence of multiple chronic conditions is higher among elderly adults [43]. These additional health conditions could also potentially affect patients’ information acquisition behaviors. To minimize the potential impacts of additional chronic conditions and unfamiliarity with internet usage, the study focuses on patients with diabetes who are 18 to 44 years old. Therefore, this study aims to investigate the model in the context of diabetes among young adults aged 18 to 44 years in the United States to understand the potentially influential roles of 2 types of online information acquisition behaviors (active seeking and incidental acquisition) and eHealth literacy on shared decision-making.

### Hypotheses

**Figure 1.**
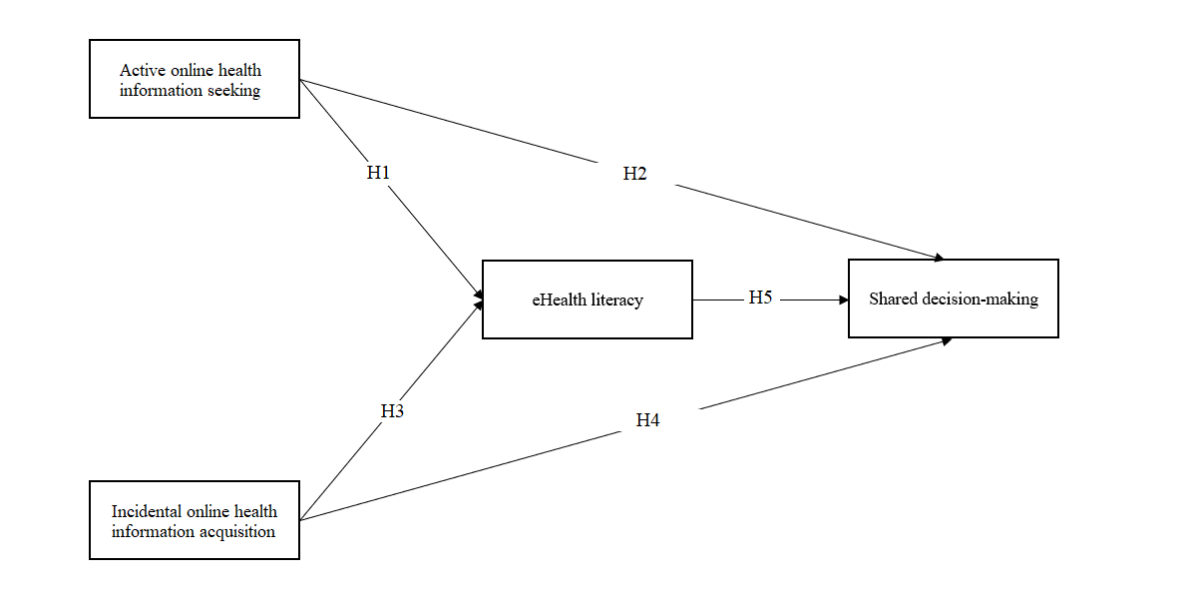
Hypothesis model.

We propose a model (Figure 1) to test the following hypotheses:

- H1: Active online health information seeking is related to eHealth literacy.
- H2: Active online health information seeking is related to shared decision-making.
- H3: Incidental online health information acquisition is related to eHealth literacy.
- H4: Incidental online health information acquisition is related to shared decision-making.
- H5: eHealth literacy is positively related to shared decision-making.

## Methods

The CHERRIES (Checklist for Reporting Results of Internet E-Surveys) guideline [44] was followed to report the study.

### Ethical Considerations

An institutional review board application for this study was reviewed by the Kent State Institutional Review Board (application number: 627). The application received an exempt approval. Informed consent was obtained from participants. An online consent form was provided on the first page of the online survey. Participants had the option to opt out on the consent form page. This study collected survey data from participants who consented. No personally identifying information was collected from participants. The data were downloaded from a password-protected Qualtrics account in an anonymous data format. The data were stored in password-protected cloud storage solutions provided by the institution. Participants received compensation from the research panel company, Centiment. They were compensated via PayPal, with an option to donate survey earnings to a nonprofit organization of their choice.

### Participants

This study is a part of a larger research project aimed at studying online health information acquisition among patients with diabetes in the United States. To achieve a statistical power of 80% at a .05 significance level to detect a medium effect (0.3) for the statistical analysis of the overall project, a power analysis was conducted, which determined that the recommended sample size is 489. After receiving institutional review board approval, we recruited participants through a research panel company, Centiment, who self-reported that they had been diagnosed with diabetes, were aged 18 to 44 years, and were living in the United States. Centiment mainly recruits survey participants through various social media sites. Its sampling process matches the national distribution of gender and age. An online questionnaire, which took about 12 minutes to complete, was distributed to participants through Qualtrics. The survey was distributed to eligible participants by Centiment from March 15 to March 28, 2023. As each item in the survey was required to be answered, there are no missing data. A total of 558 valid responses were collected.

### Measures

The shared decision-making instrument was based on a 9-item shared decision-making questionnaire [45]. A 5-point Likert scale (ranging from strongly disagree to strongly agree) was used to determine participants’ agreement with each item. An average score was calculated to reflect each participant’s overall shared decision-making involvement. eHealth literacy was measured based on Norman and Skinner’s eHealth Literacy Scale [17]. A 5-point Likert scale (ranging from strongly disagree to strongly agree) was used to determine participants’ agreement with each item. An average score was calculated to reflect every participant’s overall eHealth literacy level. Active online health information seeking was measured based on the active information-seeking instrument from Kelly et al [46]. A 5-point Likert scale (from strongly disagree to strongly agree) was used to determine participants’ agreement with each item. An average score was calculated to reflect each participant’s overall active online health information-seeking level about diabetes. Incidental online health information acquisition was measured based on the passive information-seeking instrument from Kelly et al [46]. A 5-point Likert scale (ranging from strongly disagree to strongly agree) was used to determine participants’ agreement with each item. An average score was calculated to reflect each participant’s overall incidental online health information acquisition level about diabetes. For detailed survey items, see Multimedia Appendix 1.

### Data Analysis

First, descriptive analyses were conducted to understand the general characteristics of the study sample. Second, bivariate analyses including Pearson’s correlation, 2-tailed *t* test, and Kruskal-Wallis test were used to explore bivariate relationships. Then, a partial least squares structural equation model (PLS SEM) was performed to test the proposed hypotheses. Bootstrapping was also conducted afterward. All analyses were performed in R. The R package SEMinR [47] was used for PLS SEM analysis and bootstrapping.

## Results

The valid sample had an average age of 35.91 (SD 6.04) years. Among participants, 46.59% (n=260) were men, 52.15% (n=291) were women, and 1.25% (n=7) of the participants reported their gender as other. In terms of race, 57.53% (n=321) of the participants were White or Caucasian, 15.59% (n=87) were Black or African American, 11.65% (n=65) were Hispanic and/or Latinx, and 15.23% (n=85) were categorized as other. Regarding education level, about 31% (n=173) of the participants reported having no college experience, while 69% (n=385) reported having college experience. Regarding marital status, about 45.52% (n=254) of the participants were married, and 32.26% (n=180) of them are single, never married, followed by living as married or living with a romantic partner (n=76, 13.62%), divorced (n=39, 6.99%), and widowed (n=9, 1.61%). The average general health score of the sample is 2.93, with an SD of 1.07 and a range from 1 to 5. Bivariate analyses including Pearson correlation, 2-tailed *t* test, and Kruskal-Wallis test were conducted to identify possible relationships between demographic variables and eHealth literacy and shared decision-making, respectively. Notably, education level was significantly related to both eHealth literacy and shared decision-making. Specifically, compared to those who had no college experience, those with college experience demonstrated a higher level of eHealth literacy (group mean for no college experience=3.96, group mean for “with college experience”=4.18; *P*<.001) and shared decision-making (group mean for “no college experience”=3.84, group mean for “with college experience”=4.01; *P*=0.004). Moreover, the level of general health of the sample was negatively associated with eHealth literacy (*r*=–0.18; *P*<.001) and shared decision-making (*r*=–0.22; *P*<.001). That is, those in the sample who had poorer general health in the sample tended to maintain a higher level of eHealth literacy and participate in shared decision-making at a higher level. Detailed information about the sample is shown in Table 1.

**Table 1.** Demographic information of the sample (N=558).

Demographic characteristics	Values	eHealth literacy, *P* value	Shared decision-making, *P* value
Age (y), mean (SD)	35.91 (6.04)	.10	.57
Gender, n (%)		.12	.11
Men	260 (46.59)		
Women	291 (52.15)		
Other	7 (1.26)		
Race, n (%)		.06	.08
Black or African American	87 (15.59)		
Hispanic and/or Latinx	65 (11.65)		
White or Caucasian	321 (57.53)		
Other	85 (15.23)		
Education, n (%)		<.001	.004
No college experience	173 (31)		
With college experience	385 (69)		
Marital status, n (%)		.17	.28
Married	254 (45.52)		
Living as married or living with a romantic partner	76 (13.62)		
Divorced	39 (6.99)		
Widowed	9 (1.61)		
Single, never been married	180 (32.26)		
General health, mean (SD)	2.93 (1.07)	<.001	<.001

Pearson correlation was used to identify relationships between major independent variables and outcome variables. Mean, SD, and Cronbach *α* for each instrument are also reported. The sample reported an average score of 4.00 for shared decision-making (SD 0.84, *α*=0.91), 3.97 for active online health information seeking (SD 0.86, *α*=0.78), 4.28 for incidental online health information acquisition (SD 0.80, *α*=0.85), 4.11 for eHealth literacy (SD 0.69, *α*=0.89). Cronbach α for each instrument is over 0.70, which indicates a good fit. Table 2 discusses detailed results.

**Table 2.** Correlation between continuous variables.

Variables	1	2	3	4
1. Active online health information seeking				
*r* value	1			
*P* value	—^a^			
2. Incidental online health information acquisition				
*r* value	0.65	1		
*P* value	<.001	—		
3. eHealth literacy				
*r* value	0.41	0.45	1	
*P* value	<.001	<.001	—	
4. Shared decision-making				
*r* value	0.41	0.37	0.53	1
*P* value	<.001	<.001	<.001	—
Mean (SD; range)	3.97 (0.78; 1‐5)	4.27 (0.78; 1‐5)	4.11 (0.69; 1‐5)	4.00 (0.84; 1‐5)
Cronbach *α*	0.79	0.83	0.89	0.91

a Not applicable.

In order to test the model, we performed the Henze-Zirkler multivariate normality test on the data. The results (*P*<.05) indicated that there is not enough evidence to suggest the variables follow a multivariate normal distribution. We then decided to use PLS SEM to test the model because it does not assume data normality and it is more suitable for analyzing the data [48].

To perform the PLS SEM analysis, we first assessed the reflective measures of the study through item loadings, internal consistency reliability, average variance extracted (AVE), and discriminant validity. The general criteria for indicating validity for each index are as follows: (1) all item loadings need to exceed 0.70, (2) composite reliability values need to be between 0.70 and 0.95, (3) AVE values need to exceed 0.50, and (4) discriminant validity values need to be lower than 0.85. According to the results, only 2 items (eHealth literacy item 7 and shared decision-making item 1) had loadings lower than 0.70, while all other item loadings exceeded the threshold. These two items were kept in the analysis for two reasons: (1) all other indices (internal consistency reliability, AVE, and discriminant validity) indicated that the construct is acceptable [49], and (2) for items with a loading between 0.4 and 0.7, it is recommended to remove them only when their removal would result in composite reliability and AVE increasing to meet the thresholds [50]. All other indices for all constructs met the criteria mentioned above. Table 3 shows factor loadings and reliability indices, and Table 4 shows discriminant validity.

**Table 3.** Factor loadings for the partial least squares structural equation modeling (PLS SEM) analysis.

Variables	Loading	Alpha	Composite reliability	AVE^a^
Active online health information seeking		0.791	0.856	0.544
1	0.716			
2	0.744			
3	0.738			
4	0.721			
5	0.768			
Incidental online health information acquisition		0.832	0.899	0.748
1	0.835			
2	0.889			
3	0.870			
eHealth literacy		0.891	0.913	0.568
1	0.757			
2	0.761			
3	0.778			
4	0.804			
5	0.792			
6	0.718			
7	0.694			
8	0.715			
Shared decision-making		0.910	0.926	0.586
1	0.576			
2	0.708			
3	0.730			
4	0.802			
5	0.812			
6	0.770			
7	0.821			
8	0.842			
9	0.793			

a AVE: average variance extracted.

**Table 4.** Discriminant validity.

Variables	1	2	3	4
1. Active online health information seeking				
2. Incidental online health information acquisition	0.810			
3. eHealth literacy	0.489	0.526		
4. Shared decision-making	0.484	0.397	0.586	

**Figure 2.**
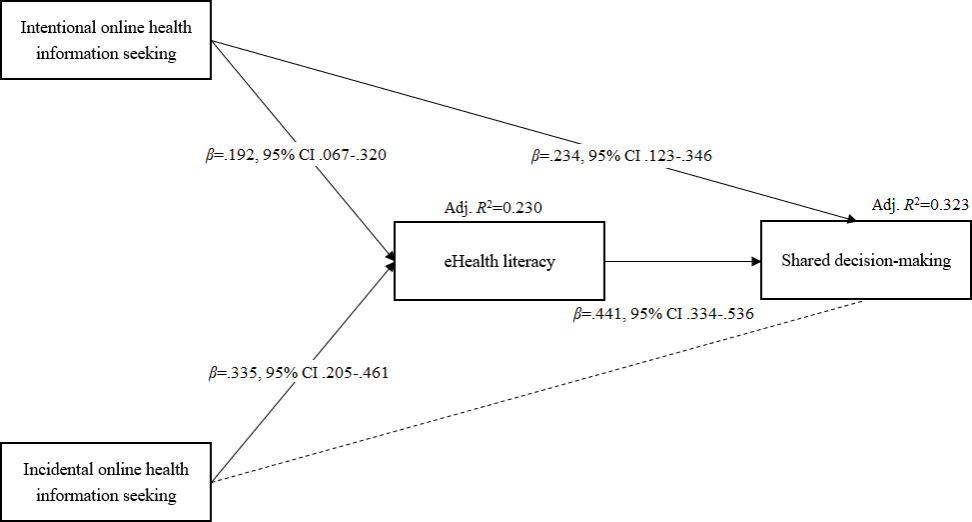
Model results of partial least squares structural equation modeling (PLS SEM).

Next, we examined the hypotheses using PLS SEM to estimate the coefficients of each path in the model and performed bootstrapping to estimate a CI to determine the statistical significance of the coefficients. The results for each path are shown in Table 5 and Figure 2.

**Table 5.** Estimations for each path of the partial least squares structural equation modeling (PLS SEM) analysis (after bootstrapping).

Variables	eHealth literacy^a^* β* (95% CI)	Shared decision-making^b^* β* (95% CI)
Active online health information seeking	.192 (.067 to .320)	.234 (.123 to .346)
Incidental online health information acquisition	.335 (.205 to .461)	−.007 (−.132 to .113)
eHealth literacy	—^c^	.441 (.334 to .536)

a Adjusted *R*2: 0.230.

b Adjusted *R*2: 0.323.

c Not applicable.

Specifically, both active online health information seeking (*β*=.192, 95% CI .067-.320) and incidental online health information acquisition (*β*=.335, 95% CI .205-.461) were positively associated with eHealth literacy; thus, H1 and H3 were supported. Active online health information seeking (*β*=.234, 95% CI .123-.346) was positively associated with shared decision-making; thus, H2 was supported. eHealth literacy was positively associated with shared decision-making (*β*=.441, 95% CI .334-.536); thus, H5 was supported. There is not enough evidence to suggest that incidental online health information acquisition was directly associated with shared decision-making; thus, H4 was not supported. In other words, while both active online health information seeking and incidental online health information acquisition were positively associated with eHealth literacy, mediation effects from eHealth literacy on shared decision-making were different for these 2 types of online health information acquisition behaviors. The data show that eHealth literacy partially mediated the relationship between active online health information seeking and shared decision-making and fully mediated the relationship between incidental online health information acquisition and shared decision-making.

Additionally, the value of the adjusted *R*^2^ for eHealth literacy (Adj. *R*^2^=0.230) indicated that active online health information seeking and incidental online health information acquisition contributed to about 23% of the variance in eHealth literacy. Meanwhile, the value of the adjusted *R*^2^ for shared decision-making (Adj. *R*^2^=0.323) suggested that active online health information seeking, incidental online health information acquisition, and eHealth literacy contributed to about 32.3% of the variance in shared decision-making. These results further illustrate that the model explains a significant amount of the variance in eHealth literacy and shared decision-making.

## Discussion

### Main Findings

This study explored the association between 2 types of online health information acquisition behavior and shared decision-making and investigated the role of eHealth literacy within the process among US patients with diabetes aged 18 to 44 years. Unlike previous studies, this study investigated both active information seeking and incidental information acquisition rather than only concentrating on active information seeking alone or merging the 2 types of information acquisition together. Multiple hypotheses were proposed within the model and examined through a PLS SEM approach. Descriptive analysis results suggest that the prevalence of incidental online health information acquisition is high among the target population. This aligns with other scholars’ arguments that younger people tend to incidentally acquire information through encountering it rather than actually searching for it [51]. This suggests that it is necessary and urgent to raise awareness among information and health professionals of active information-seeking behavior in young people with diabetes and develop appropriate strategies to distribute accurate and timely health information and messages to them. For instance, information professionals and public health agencies could collaborate to increase the promotion of structured and accurate health information through popular online platforms and enhance the readability and interactivity of such information.

The results from bivariate analyses indicated that education is a key factor related to eHealth literacy and shared decision-making. Those who have college experience tend to have a higher level of eHealth literacy and shared decision-making. This finding echoes numerous previous studies and supports the conclusion that it is crucial to increase education levels among the population to help improve population health and that education can be viewed as a form of health policy to enhance quality of life [52-54]. Additionally, negative relationships between general health level and eHealth literacy, as well as general health level and shared decision-making are worth noting. Compared to people with better general health, those with poorer general health have a greater need for health information. As cognitive need is a factor that motivates users to perform information behavior [55], it follows that a greater need for health information could lead to more health information acquisition online. With more frequent online health information acquisition, those with poorer health could get more exposure to health information via the internet. As previously illustrated, frequent exposure to online health information can be related to higher levels of eHealth literacy [30,31]. Furthermore, people with poor health might have a greater need to improve their health status, and shared decision-making is an effective step for them to take to improve their health situation [56], while people with good or excellent health may have less need to participate in shared decision-making. Therefore, these 2 negative relationships observed within our sample are important to highlight as opportunities for health professionals to develop targeted communication strategies.

The results from our model testing using PLS SEM indicated that eHealth literacy mediated the relationship between both types of online health information acquisition behavior and shared decision-making differently. Specifically, active online health information seeking was associated with both eHealth literacy and shared decision-making; therefore, eHealth literacy partially mediated the relationship between active online health information seeking and shared decision-making. Incidental online health information acquisition was only associated with eHealth literacy and shared decision-making; therefore, eHealth literacy fully mediated the relationship between incidental online health information acquisition and shared decision-making. The results show that eHealth literacy is a crucial mechanism that connects online health information acquisition to shared decision-making. This suggests 2 interesting paths that can increase shared decision-making: increasing one’s level of active online health information seeking or increasing individual eHealth literacy skills. These results partially align with previous research, which found that active online information seeking is a crucial factor related to shared decision-making [10], and its role can be enhanced through eHealth literacy [32]. Although there are previous studies that indicate that incidental online information acquisition may be related to other types of decision-making [57,58], no other study has examined the relationship between incidental online information acquisition and shared decision-making. This study addresses that gap by exploring the role that incidental online health information acquisition plays in shared decision-making. However, it is worth noting that because this study measured shared decision-making in a general way rather than focusing on diabetes-related shared decision-making, the specific relationship between diabetes online information acquisition and shared decision-making engagement on diabetes still needs further investigation.

The results also suggest that in addition to the active health online information seeking, incidental online health information acquisition could also help an individual search for, evaluate, and use online health information (eHealth literacy). Through an increased level of eHealth literacy, those who have a higher level of incidental online health information acquisition may increase their engagement in shared decision-making. These 2 paths also highlight differences between active online health information seeking and incidental online health information acquisition, while still demonstrating the benefits of incidental online health information acquisition. With the broad use of social media and other internet technologies, incidental exposure to different types of online information is high [58,59]. Thus, it is possible that there are benefits to incidental online information acquisition more generally, beyond the health context.

### Implications

Practically, the results of this study can contribute to the continuing development of health communication strategies and the modification of health information training programs for patients with diabetes. First, this study highlights the importance for the government and related agencies to continue enhancing health education policies and increasing health education levels among the population [60,61]. Second, in order to promote shared decision-making among patients with diabetes, it is necessary to improve information seekers and users’ eHealth literacy and the credibility of online information that information seekers may incidentally pay attention to. Proper training programs or workshops from information institutions and health agencies could be provided to patients with diabetes to help them identify high-quality health information resources and gain useful online information-seeking skills amid an ever-changing online environment. The need for this training is becoming increasingly urgent, as recent research points to a deterioration in the effectiveness of search engines in returning relevant results [62]. Further, since the shared decision-making process involves a 2-way communication process, it is also necessary to raise awareness of how to improve the communication skills of health professionals. Specific technologies, such as clinical decision support tools, may be harnessed to support medical professional or patient communication [63,64]. To improve the equity of health care services, strategies to help reduce complicit bias, improve communication effectiveness, and enhance health information presenting skills are also highly paramount [65,66]. Health agencies and medical schools can help prepare both patients and health providers with the information and skills to be able to maximize the benefits of the shared decision-making process.

Moreover, as various researchers have illustrated, similar concepts such as artificial serendipity can be refined and shaped within digital environments [67,68]. It is also crucial for health agencies to use emerging technologies to improve the quality of health information that would be recommended or exposed to patients with diabetes incidentally. According to Hassoun et al [51], younger generations tend to start their information journey by scanning and encountering online and then interpret and consume information with other online peers together as a social group. It is essential to keep an eye on social media platforms to monitor the quality of health information. As many researchers have pointed out, social media algorithms manipulate the content that users read and encounter online [69,70]. Thus, it is crucial for the information industry to make an effort to deliver accurate health information to the public in support of their decision-making process and encourage positive health behaviors.

As high-speed internet access remains unreliable in parts of the United States, it is also important to close the gap in access to and use of the internet, especially among patients with diabetes. Community health programs and local institutions can play an important role in this effort. For example, providing free access to computers within local public libraries and organizing tutorial programs to help those not familiar with the online environment could bridge the information gap for patients with diabetes with limited internet access. Moreover, since the number of patients with diabetes is large and patients are not all the same, for instance, older adults tend to be less familiar with digital technologies, it is necessary to provide customized training programs to address issues for different groups of patients with diabetes.

### Limitations and Future Directions

There are several limitations within this study. First, it used cross-sectional data; therefore, the results can only contribute to association instead of causation effects. Second, the instrument of each variable is self-reported. There might be potential bias in the responses. For instance, one can self-report with great confidence on finding health information online but actually have false perceptions about the accuracy of online health information sources. Third, the study examined only online information acquisition, while other ways of information acquisition, such as interpersonal information acquisition, were not investigated. Fourth, this study did not collect information from participants regarding the type of diabetes they were diagnosed with. Patients with different types of diabetes (type 1 diabetes, type 2 diabetes, or gestational diabetes) may focus on different types of information from various sources. Fifth, the study investigated only adult patients with diabetes who are under 45 years. Since younger adults tend to use online technologies more frequently compared to older adults, it is important to note that the generalizability of the study findings is limited. Moreover, the sampling process only matched national distribution on gender and age. Matching more key demographic characteristics, such as race, ethnicity, and region, could further enhance the quality of the collected data. Additionally, other important social determinants were not included in this study, such as food insecurity, safety, homelessness, drug use, and comorbidities. These factors may also be related to the information behaviors of patients with diabetes.

Future studies could collect longitudinal data to further examine causation effects. The model could also be modified and extended by including more tailored measurements to other health contexts or populations within other age groups. It would also be useful for future studies to separately measure patients with type 1, type 2, and gestational diabetes to examine whether there are differences in their information acquisition behaviors and related impacts. Furthermore, it would be more appropriate for future studies to contextualize general measurements within the study context to collect context-specific data.

## Supplementary material

10.2196/86137 Multimedia Appendix 1 Survey items for key variables.

## Abbreviations

AVE: average variance extracted
CHERRIES: Checklist for Reporting Results of Internet E-Surveys
PLS SEM: partial least squares structural equation modeling
