# Supplementary material for: Relationship Between Online Health Information Acquisition and Shared Decision-Making Among Patients With Diabetes: Cross-Sectional Survey Study
Source: J Med Internet Res. 2026 Apr 30;28:e86137. doi: 10.2196/86137 (PMC13132485; doi:10.2196/86137)
Supplement: Multimedia Appendix 1 [file jmir-v28-e86137-s001.docx]

**Appendix**

*Survey Items for Key Variables*

| Variable | Item |
| --- | --- |
| Active online health information seeking | I have asked my doctor for information about diabetes online |
|  | I have searched for information on diabetes online |
|  | I have talked to friends about diabetes online |
|  | I watch for breaking news on diabetes online |
|  | I have looked for new information on diabetes online |
| Incidental online health information acquisition | I listen when I hear news about diabetes online |
|  | I read news about diabetes online when I come across it |
|  | Information about diabetes online catches my attention |
| eHealth literacy | I know how to find helpful health resources on the Internet |
|  | I know how to use the Internet to answer my health questions |
|  | I know what health resources are available on the Internet |
|  | I know where to find helpful health resources on the Internet |
|  | I know how to use the health information I find on the Internet to help me |
|  | I have the skills I need to evaluate the health resources I find on the Internet |
|  | I can tell high quality from low quality health resources on the Internet |
|  | I feel confident in using information from the Internet to make health decisions |
| Shared decision-making | My doctor made clear that a decision needs to be made |
|  | My doctor wanted to know exactly how I want to be involved in making the decision |
|  | My doctor told me that there are different options for treating my medical condition |
|  | My doctor precisely explained the advantages and disadvantages of the treatment options |
|  | My doctor helped me understand all the information |
|  | My doctor asked me which treatment option I prefer |
|  | My doctor and I thoroughly weighed the different treatment options |
|  | My doctor and I selected a treatment option together |
|  | My doctor and I reached an agreement on how to proceed |
